# Correlates of Biochemical Markers of Bone turnover among Post-Menopausal Women

**DOI:** 10.31729/jnma.3604

**Published:** 2018-08-31

**Authors:** Bikram Khadka, Mohan Lal Tiwari, Ravi Gautam, Binod Timalsina, Nandu Prasad Pathak, Krishna Kharel, Shanta Sharma, Dilaram Acharya

**Affiliations:** 1 Department of Biochemistry, Devdaha Medical College and Research Institute, Devdaha, Rupandehi, Nepal; 2 Department of Internal Medicine, Devdaha Medical College and Research Institute, Devdaha, Rupandehi, Nepal; 3 Department of Occupational Health, College of Bio-Medical Sciences, Daegu Catholic University, Gyeongsan 38430, Korea; 4 Department of Anatomy, Devdaha Medical College and Research Institute, Devdaha, Rupandehi, Nepal; 5 Department of Internal Medicine, Lumbini Zonal Hospital, Rupandehi, Butwal, Nepal; 6 Department of Orthopedic Surgery, Tilottama Hospital, Rupandehi, Butwal, Nepal; 7 Deapartment of Community Medicine, Devdaha Medical College and Research Institute, Devdaha, Rupandehi, Nepal; 8 Department of Preventive Medicine, College of Medicine, Dongguk University, Gyeongju, Republic of Korea; 9 Department of Community Medicine, Devdaha Medical College and Research Institute, Devdaha, Rupandehi, Nepal

**Keywords:** *biochemical markers*, *bone turnover*, *osteoporosis*, *post-menopause*

## Abstract

**Introduction:**

Bone turnover leading to osteoporosis and poor quality of life is common during postmenopausal period. Study of bone turnover markers that contribute to non-invasive assessment of bone-metabolic disorders holds an important area of research in low income country like Nepal. This study aimed to examine the correlates of bone turnover markers in post-menopausal women in tertiary level of health care center of Nepal.

**Methods:**

A hospital-based cross-sectional study conducted during the period of November 2016 to December 2017 among 354 women. Blood samples for calcium, inorganic phosphorus, alkaline phosphatase and vitamin D were collected and analyzed using a validated and calibrated tools. Data were analyzed using Statistical Package for the Social Sciences software version 20.

**Results:**

Mean+Standard deviation of age of post-menopausal women was significantly higher compared to pre-menopausal women (post-menopausal women, (57.98±8.08) vs. pre-menopausal, (31.35±5.83), (P<0.001). Selected biochemical markers of bone-turnover such as alkaline phosphatase levels were significantly higher with year since menopause (P<0.001), whereas serum calcium, and vitamin D were decreasing with year since menopause among post-menopausal women. In addition, calcium and vitamin D were significantly negatively correlated with year since menopause (P<0.01) while body mass index, inorganic phosphorus and alkaline phosphatase were significantly positively correlated with year since menopause (P<0.01).

**Conclusions:**

Our study revealed that body mass index, inorganic phosphorus and alkaline phosphatase positively correlated with year since menopause while calcium and vitamin D were negatively correlated suggesting for a medical supervision of hormonal changes and periodic dosing of calcium and vitamin D among post-menopausal women to reduce the problem of bone health.

## INTRODUCTION

Bone is a dynamic tissue that is being constantly remodeled throughout life in a balanced way between formation and resorption is disturbed leading to osteoporosis during menopausal period.^1,2^ Osteoporosis is one of the most common morbid conditions in post-menopausal life that occur due to the impact of hormonal changes, markedly reduces the quality of life.^3–5^

Study of bone turnover markers contributes to non-invasive assessment of bone-metabolic disorders, although bone density measurements and biopsy has already been practiced. Biochemical markers reflecting the rate of bone turnover holds greater importance in predicting the rate of bone loss in post-menopausal women.^6,7^

With this study background, this study aimed to examine the association of bone turnover markers in post-menopausal women in tertiary level of health care center of Nepal.

## METHODS

This is a cross-sectional study conducted at Devdaha Medical College and Research Institute employing a systematic random sampling technique during the period of November 2016 to December 2017. The research proposal was approved by Institutional Review Committee (IRC) of Devdaha Medical College and Research Institute. We obtained the written informed consent from every participants enrolled.

The sample size of this study was calculated on the basis of previously published paper which reported the prevalence of 33.3% post-menopausal women were osteoporotic^8^ using the formula, n= z^2^pq / e^2^


$$\begin{array}{l} =\;{\left(1.96\right)}^{2}\;\times \;0.333\;\times \;0.667\;/\;0.0025\\ =\;341.16 \end{array}$$

(where, z= 1.96 at 95% of Confidence Interval (CI)

P= prevalence of osteoporotic women=33.33%
q= (1-p)

and e= permissible error at 5% with degree of assurance as 95% confidence level).

We enrolled 354 women, 177 were pre-menopausal (23–43 years old) and the rest were post-menopausal women (>45 years old). Variables like age, sex and years since menopause (YSM) were obtained using comprehensive questionnaire filled through direct interview from the study participants. Anthropometric measurements like weight, height and body mass index (BMI) were measured. BMI of each individual was calculated as their weight (kg) divided by the square of their height (m^2^).

Five milliliter of venous blood collected in a gel tube was allowed to clot, and centrifuged at 3000 RPM for 10 minutes for estimation of serum calcium, alkaline phosphatase (ALP), serum phosphorus and vitamin D (25-OH Vitamin D). Serum calcium and inorganic phosphorus level were estimated using colorimeter by Biuret and Modified Gomorri's method respectively, while alkaline phosphatase activity was determined using kinetics method by Earba Chem 5 V3 semi-automated chemistry analyzer. Enzyme linked immunosorbent assay kits was used for quantitative determination as serum vitamin D levels.

Study participations with known case of osteoporotic features, renal insufficiency, liver dysfunction, cirrhosis, hormonal replacement therapy, calcium and vitamin D supplementation within the past 6 months, on the steroid treatment for more than 6 months and pre-menopausal women with irregular menstrual cycles were excluded from the study.

Continuous variables with normal distribution are presented as mean and standard deviation (SD) using independent t-test to compare the clinical characteristics between pre-and post-menopausal women, while One-way analysis of variance (ANOVA) was employed to assess the independent association of clinical characteristics to compare means of different age groups of post-menopausal women. Moreover, persons correlation test was also employed to assess the correlation between different study variables. All statistical significance are reported at P<0.05. All statistical analyses were performed using Statistical Package for the Social Sciences (SPSS) software version 20.

## RESULTS

Clinical characteristics of pre-menopausal and post-menopausal women have been detailed (Table 1). Mean±Standard deviation (SD) of age of Post-menopausal women was significantly higher compared to pre-menopausal women (post-menopausal women, (57.98±8.08) vs. pre-menopausal, (31.35±5.83), (P<0.001)). BMI, Inorganic Phosphorus, ALP were also significantly higher in post-menopausal women than pre-menopausal women (P<0.001), while Calcium, and Vitamin D were significantly lower in post-menopausal women compared to pre-menopausal women (P<0.001).

**Table 1:** Clinical characteristics of the Pre-menopausal and Post-menopausal women (n = 354).

Variables	Pre-menopausal (control)	Post-menopausal (Case)	P
	Mean ±SD	Mean ±SD	
Age (years)	31.35±5.83	57.98±8.08	0.001^*^
BMI (kg/m^2^)	25.29±3.79	28.19±4.46	0.001^*^
Calcium (mg/dl)	9.38±0.43	8.52±0.54	0.001^*^
Inorganic phosphorus (mg/dl)	3.34±0.46	3.98±0.34	0.001^*^
ALP (U/L)	81.2±18.22	114.62±33.45	0.001^*^
25-OH Vitamin D (ng/ml)	27.86±3.73	20.58±1.98	0.001^*^

* *Statistically significant at P<0.001*

Differentials of clinical characteristics among post-menopausal by year since menopause (YSM) has been demonstrated (Table 2). Serum ALP levels were significantly higher with YSM (P<0.001) whereas serum calcium, and vitamin D were decreasing with YSM among post-menopausal women.

**Table 2:** Comparison of YSM with BMI, serum calcium, serum inorganic phosphorus, serum alkaline phosphatase and Vitamin D level among post-menopausal women (n=177).

YSM (years)	1–5years (n = 58)	6–10 years (n=83)	11–15 years (n=32)	> 15years (n=4)	P
BMI (kg/m^2^)	28.80±4.52	27.71±4.35	28.27±4.67	28.64±3.97	0.34
Calcium (mg/dl)	8.55±0.52	8.57±0.49	8.57±0.68	8.27±0.78	0.01^*^
Inorganic phosphorus (mg/dl)	3.98±0.33	3.96±0.38	3.77±0.39	4.10±0.08	0.87
ALP (U/L)	110.27±24.39	115.88±38.13	116.23±36.13	138.00±0.00	0.00^*^
25-OH Vitamin D (ng/ml)	20.66±2.00	20.75± 0.87	20.43±1.89	17.25±0.50	0.04^†^

* *Statically significant at the 0.05 level (2-tailed)*

† *statistically is significant at the 0.01 level (2- tailed)*

Table 3 shows correlation of selected bone turnover markers of post-menopausal women. Calcium and Vitamin D were significantly negatively correlated with Year Since Menopause (YSM) (P<0.01) while BMI, Inorganic phosphorus and ALP were significantly positively correlated with Year Since Menopause (YSM) (P<0.01)

**Table 3:** Correlation of different variables among post-menopausal women. (n=177)

Variables	BMI (kg/m^2^)	Calcium (mg/dl)	Inorganic phosphorus	ALP (U/L)	25-OH Vitamin D (ng/ml)	YSM (years)
BMI (kg/m^2^)	–	−0.177^*^	0.287^*^	0.134^†^	−0.284^*^	0.296^*^
Calcium (mg/dl)	−0.177^*^	–	−0.573^*^	−0.515^*^	0.557^*^	−0.540^*^
Inorganic phosphorus (mg/dl)	0.287^*^	−0.573^*^	–	0.382^*^	−0.446^*^	0.451^*^
ALP (U/L)	0.134^†^	−0.515^*^	0.382^*^	–	−0.419^*^	0.462^*^
25-OH Vitamin D (ng/ml)	−0.284^*^	0.557^*^	−0.446^*^	−0.419^*^	–	−0.638^*^
YSM (years)	0.296^*^	−0.540^*^	0.451^*^	0.462^*^	−0.638**	–

* *Correlation is significant at the 0.05 level (2-tailed)*

† *Correlation is significant at the 0.01 level (2- tailed)*

## DISCUSSION

Health problems among post-menopausal women are numerous, which not only give rise to economic burden but also markedly reduces the quality of life. In this study, we found marked differences of some bone turnover markers in post-menopausal women compared to pre-menopausal women. Similar to other studies, we found BMI, Inorganic Phosphorus and ALP were significantly higher in post-menopausal women than pre-menopausal women, while Calcium and Vitamin D were significantly lower in post-menopausal women.^9–11^ Converse to our study, previous study found that serum calcium level of post-menopausal participants was significantly higher than pre-menopausal women.^2^ Differences might be attributable to sampling and measurement variation and whether post-menopausal women were receiving the supplementary therapy of vitamin D and calcium.

Serum alkaline phosphatase is an important enzyme and commonly used bone turnover marker which plays major role in bone formation and mineralization. Total serum ALP pool is composed of different dimeric isoforms which originate from various tissues like liver, bone, intestine, spleen, kidney and placenta of which approximately 50% of total serum ALP is derived from liver in the adults with normal liver function and 50% arises from bone.^12^ Bone specific alkaline phosphatase (BAP) is known to be sensitive marker for bone turnover and also in postmenopausal osteoporosis.^13,14^ In addition, Romagnoli et. al. and Woitge et. al. reported that total ALP is more sensitive index of bone formation than BAP.^15,16^

Other major findings of our study is that serum ALP levels were significantly higher with late years since menopause (>10 YSM) and serum calcium, and vitamin D were decreasing with YSM among post-menopausal women. However, contrary to these findings higher serum calcium and total ALP activity have been demonstrated in early postmenopausal women (≤10 YSM) compared to late postmenopausal women (>10 YSM).^17,18^ The responsible factors for disparity in the levels of parameters of bone turnover may due to heterogeneity of older adults, difference in body physiology and metabolism, their dietary and nutrition habits and their unique rate of aging. In addition to these, Calcium and Vitamin D were significantly negatively correlated with YSM, while BMI, inorganic phosphorus and ALP were significantly positively correlated with YSM suggesting that bone turnover markers such BMI, inorganic phosphorus, ALP increases with increasing number of YSM and calcium and vitamin D decreases correspondingly with increased menopausal duration. The condition might be evident due to deficiency of estrogen hormone since aging and menopause leads to reduced production of estrogen. Estrogen deficient state induces synthesis of cytokines by osteoblasts, monocytes, and T-cells and thereby stimulates bone resorption by increasing osteoclastic activity which has been implicated in the increased ALP levels of post-menopausal women.^2^

Vitamin D is another critical factor modulating bone growth and turnover. Vitamin D deficiency and related diseases are prevalent in post-menopausal women.^19^ Normally vitamin D is responsible for maintaining a proper calcium level mainly by promoting the intestinal calcium absorption. Several studies reported that reduced vitamin D level in post-menopausal women.^11,20^ Reduced vitamin D in post-menopausal women is known to be influenced by age related decrease in the cutaneous synthesis of vitamin D, decline renal production of the hormone calcitriol, poor intestinal sensitivity to vitamin D absorption, inadequate sun exposure and hyperparathyroidism increases the bone resorption and accelerates the bone loss.^21–23^ Reduction of vitamin D is also associated with obesity, probably because of lipophilic structure of vitamin D which induces its segregation in the adipose tissue.^24^ Vitamin D and calcium supplements may be beneficial for elderly and menopausal women in this regard as vitamin D and calcium theoretically increases the bone mineralization and decreasing hyperparathyroidism and bone turnover.

We note some of the important strengths of this study. First, we identified some of the important selected biochemical markers of bone turnover among post-menopausal women in a tertiary level health care setting in Nepal. Second, this study might be useful for health authority to design evidence based preventive strategies. However, there are some of the significant limitations of this study. First, this study could not include the most sensitive markers of bone turnover like Dual Energy X-ray Absorptiometry (DEXA), N-terminal Telopeptide (NTx), bone mineral density measurements, BAP, urinary hydroxyproline and osteocalcin. Second, small sample size and limited area of Rupandehi district of Nepal could not allow us to generalize the study findings. Third, the information which affects the bone mineral metabolism like caloric intake, household wealth and household food insecurity were not assessed in this study. Further studies may be needed to understand these specific limitations.

## CONCLUSIONS

Our study revealed that BMI, Inorganic phosphorus and ALP positively correlated with year since menopause while calcium and vitamin D were negatively correlated. Additionally, serum alkaline phosphatase levels were significantly higher, and calcium and vitamin D were significantly lower among older menopausal women. This study suggests for a medical supervision of hormonal changes and periodic dosing of calcium and vitamin D among post-menopausal women to reduce the problem of bone health. Further, studies covering wide range of bone markers in large population to support the generalizability of the study are needed.

## References

[1] Jagtap VR, Ganu JV, Nagane NS. (2011). BMD and Serum Intact Osteocalcin in Postmenopausal Osteoporosis Women. Indian J Clin Biochem..

[2] Sachdeva A, Seth S, Khosla AH, Sachdeva S. (2005). Study of some common biochemical bone turnover markers in postmenopausal women. Indian J Clin Biochem..

[3] Blumel JE, Castelo-Branco C, Binfa L, Gramegna G, Tacla X, Aracena B (2000). Quality of life after the menopause: a population study. Maturitas..

[4] Charandabi SM, Rezaei N, Hakimi S, Montazeri A, Taheri S, Taghinejad H (2015). Quality of Life of Postmenopausal Women and Their Spouses: A Community-Based Study. Iran Red Crescent Med J..

[5] Keramat A, Patwardhan B, Larijani B, Chopra A, Mithal A, Chakravarty D (2008). The assessment of osteoporosis risk factors in Iranian women compared with Indian women. BMC Musculoskelet Disord..

[6] Christiansen C, Riis BJ, Rodbro P. (1987). Prediction of rapid bone loss in postmenopausal women. Lancet.

[7] Christiansen C, Riis BJ, Rodbro P. (1990). Screening procedure for women at risk of developing postmenopausal osteoporosis. Osteoporos Int..

[8] Najam R, Huda N. (2011). Assessment of Osteoporosis in Post Menopausal Women: A Clinical Study. Nepal Journal of Obstetrics and Gynaecology..

[9] Bhattarai T, Bhattacharya K, Chaudhuri P, Sengupta P. (2014). Correlation of common biochemical markers for bone turnover, serum calcium, and alkaline phosphatase in post-menopausal women. Malays J Med Sci..

[10] Indumati V, Patil VS, Jailkhani R. (2007). Hospital based preliminary study on osteoporosis in postmenopausal women. Indian J Clin Biochem..

[11] Yasovanthi J, Karunakar KV, Manjari KS, Reddy BP, Kumar PA, Charyulu MS (2011). Association of vitamin D receptor gene polymorphisms with BMD and their effect on 1, 25-dihydroxy vitamin D3 levels in pre-and postmenopausal South Indian women from Andhra Pradesh. Clin Chim Acta..

[12] Delmas PD, Eastell R, Garnero P, Seibel M, Stepan J. (2000). The use of biochemical markers of bone turnover in osteoporosis. Osteoporos Int..

[13] Coleman R, Whitaker K, Moss D, Mashiter G, Fogelman I, Rubens R. (1988). Biochemical prediction of response of bone metastases to treatment. Br J Cancer.

[14] Duda RJ, O'Brien JF, Katzmann JA, Peterson JM, Mann KG, Riggs BL. (1988). Concurrent assays of circulating bone Gla-protein and bone alkaline phosphatase: effects of sex, age, and metabolic bone disease. J Clin Endocrinol Metab..

[15] Romagnoli E, Minisola G, Carnevale V, Scillitani A, Frusciante V, Aliberti G (1998). Assessment of serum total and bone alkaline phosphatase measurement in clinical practice. Clin Chem Lab Med..

[16] Woitge H, Seibel M, Ziegler R. (1996). Comparison of total and bone-specific alkaline phosphatase in patients with nonskeletal disorder or metabolic bone diseases. Clin Chem..

[17] Rogers A, Hannon R, Eastell R. (2000). Biochemical markers as predictors of rates of bone loss after menopause. J Bone Miner Res..

[18] Suresh M, Naidu D. (2006). Influence of years since menopause on bone mineral metabolism in South Indian women. Indian J Med Sci..

[19] Looker AC, Johnson CL, Lacher DA, Pfeiffer CM, Schleicher RL, Sempos CT. (2011). Vitamin D status: United states, 2001-2006. NCHS Data Brief.

[20] Pop LC, Shapses SA, Chang B, Sun W, Wang X. (2015). Vitamin D-binding protein in healthy pre-and postmenopausal women: relationship with estradiol concentrations. Endocr Pract..

[21] Kinyamu HK, Gallagher JC, Prahl JM, Deluca HF, Petranick KM, Lanspa SJ. (1997). Association between intestinal vitamin D receptor, calcium absorption, and serum 1, 25 dihydroxyvitamin D in normal young and elderly women. J Bone Miner Res..

[22] Veldurthy V, Wei R, Oz L, Dhawan P, Jeon YH, Christakos S. (2016). Vitamin D, calcium homeostasis and aging. Bone Res..

[23] Slovik DM, Adams JS, Neer RM, Holick MF, Potts JT (1981). Deficient production of 1, 25-dihydroxyvitamin D in elderly osteoporotic patients. N Engl J Med..

[24] Cipriani C, Pepe J, Piemonte S, Colangelo L, Cilli M, Minisola S. (2014). Vitamin D and Its Relationship with Obesity and Muscle. Int J Endocrinol.

